# Effect of Electrode Modification with Chitosan and Nafion^®^ on the Efficiency of Real-Time Enzyme Glucose Biosensors Based on ZnO Tetrapods

**DOI:** 10.3390/ma15134672

**Published:** 2022-07-03

**Authors:** Valerii Myndrul, Igor Iatsunskyi, Nataliya Babayevska, Marcin Jarek, Teofil Jesionowski

**Affiliations:** 1 NanoBioMedical Centre, Adam Mickiewicz University, 3 Wszechnicy Piastowskiej Str., 61614 Poznan, Poland; natbab@amu.edu.pl (N.B.); marcin.j@amu.edu.pl (M.J.); 2 Faculty of Chemical Technology, Institute of Chemical Technology and Engineering, Poznan University of Technology, Berdychowo 4, 60965 Poznan, Poland; teofil.jesionowski@put.poznan.pl

**Keywords:** glucose, chitosan, Nafion, ZnO tetrapods, cyclic voltammetry, chronoamperometry

## Abstract

Noninvasive, continuous glucose detection can provide some insights into daily fluctuations in blood glucose levels, which can help us balance diet, exercise, and medication. Since current commercially available glucose sensors can barely provide real-time glucose monitoring and usually imply different invasive sampling, there is an extraordinary need to develop new harmless methods for detecting glucose in non-invasive body fluids. Therefore, it is crucial to design (bio)sensors that can detect very low levels of glucose (down to tens of µM) normally found in sweat or tears. Apart from the selection of materials with high catalytic activity for glucose oxidation, it is also important to pay considerable attention to the electrode functionalization process, as it significantly contributes to the overall detection efficiency. In this study, the (ZnO tetrapods) ZnO TPs-based electrodes were functionalized with Nafion and chitosan polymers to compare their glucose detection efficiency. Cyclic voltammetry (CV) measurements have shown that chitosan-modified ZnO TPs require a lower applied potential for glucose oxidation, which may be due to the larger size of chitosan micelles (compared to Nafion micelles), and thus easier penetration of glucose through the chitosan membrane. However, despite this, both ZnO TPs modified with chitosan and Nafion membranes, provided quite similar glucose detection parameters (sensitivities, 7.5 µA mM^−1^ cm^−1^ and 19.2 µA mM^−1^ cm^−1^, and limits of detection, 24.4 µM and 22.2 µM, respectively). Our results show that both electrodes have a high potential for accurate real-time sweat/tears glucose detection.

## 1. Introduction

Real-time glucose detection is a hot topic at the moment, since continuous glucose monitoring can preserve more than 420 million people over the world from severe consequences caused by diabetes, no matter the type [1]. In addition, continuous noninvasive glucose detection (e.g., indirect detection in sweat, tears, or saliva) has the advantage over conventional glucometers that require blood sampling by painful and frequent finger-pricking [2,3,4]. At this stage, the question of a correlation between a person’s blood and sweat/tears/saliva glucose levels may arise, but thanks to recent findings, one can say that such a correlation can be found for every human’s metabolism, or that the device for real-time glucose monitoring can, at least, warn the user of critical glucose levels and the occurrence of hyperglycemia and hypoglycemia [5,6]. Glucose levels in non-invasive body fluids (e.g., sweat) are known to be in the range of 17–100 μM; therefore, transducers capable of detecting such a small amount of glucose content are required [7,8]. In this regard, transducers based on nanoscaled materials can be used, since their properties (electronic, optical, chemical, etc.) get changed even with the slightest disturbances in surroundings and, thus, can be used as a sensor response [9,10].

Among all nanoscaled materials, semiconductors are considered to be the most applicable for (bio)sensors due to their diversity, relatively low cost, and advanced properties (e.g., optical, electrical, chemical) [11,12]. Transducers made of semiconductors have been used in almost all types of (bio)sensing applications including optical [13,14], electrochemical [15], piezoelectric [16], electronic [17], etc. Out of all nanoscaled semiconductors, zinc oxide (ZnO) is one of the most important due to its biocompatibility, low toxicity, and shape diversity (thin films, spheres, rods, tetrapods, flowers, etc.) [18,19,20]. For example, thin ZnO films prepared by (ALD) atomic layer deposition have proven themselves in photoluminescence-based (PL-based) detection of Grapevine virus A-type proteins [21], while ZnO nanorods appeared promising for electrochemical glucose detection with a detection limit (LOD ~60 µM) suitable for glucose detection in non-invasive body fluids [22]. However, it is worth noting that the electrochemical glucose (or other redox species) detection is also highly dependent on the transducer, and it is essential to use one with a higher surface-to-volume ratio, and better conductivity (lower resistivity) [23,24]. In this case, according to Sulciute et al., ZnO tetrapods have an advantage over both ZnO nanospheres and ZnO nanorods because they provide a percolation path with fewer barriers, resulting in lower voltage drop [25], and possess highest active surface enabling adsorption of higher amount of bioreceptors for specific interaction with analytes. In addition, the enzymatic glucose (bio)sensor performance on ZnO-based transducers also depends on how the functionalization of ZnO surface was carried out [20].

The functionalization strategy of different transducers (including ZnO) usually requires several consequent steps to be performed. At the initial step, ZnO is treated with various cross-linking agents (e.g., glutaraldehyde) [1,26], at the second-glucose oxidase (GOx) deposition [27], and at the final-deposition of a polymer layer [28,29,30]. The last one plays an essential role, as it simultaneously performs several important functions: (i) prevents of GOx from leaking out, (ii) permits charge transfer through it, and (iii) reduces interference by ionic substances [31]. These polymers can be either synthetic or organic. Nafion is the most widely used synthetic polymer [32,33], and chitosan is the most commonly used among biopolymers [34,35,36]. Given that they have different physicochemical properties, they will affect the efficiency of the electrochemical glucose detection process in different ways. Therefore, it is important to investigate the glucose detection parameters on ZnO TPs using two different functionalization strategies based on Nafion and chitosan. This would shed some light on which polymer is better suited for such applications, this would improve different detection performances.

In this study, ZnO TPs were used for electrochemical glucose detection using two different strategies of ZnO TPs functionalization with Nafion and chitosan. Samples with Nafion were denoted as ZnO TPs/Nafion, while samples with chitosan were denoted as ZnO TPs/chitosan. Besides, chemical, structural properties of the fabricated electrodes were investigated by means of Fourier-transform infrared (FTIR), Raman, XRD spectroscopies and scanning electron microscopy (SEM). In addition, the real-time amperometric determination of glucose was performed, and the main detection parameters were calculated and compared to determine which polymer had the best effect on the detection process.

## 2. Materials and Methods

Morphology/structure of the ZnO TPs was studied by scanning electron microscopy and energy dispersive spectroscopy (SEM and EDS) (JEOL, Tokyo, Japan, JSM7001F) The structural state of samples was analyzed by means of X-ray diffraction (XRD) (PANalytical, Malvern, UK, X’pert3pro MPD diffractometer) working with a Cu lamp (λ = 1.5418 Å). Raman spectrum was measured by means of a Renishaw micro-Raman spectrometer with a confocal microscope. Electrochemical measurements (cycling voltammetry and chronoamperometric glucose detection) were performed using GAMRY 620 potentiostat (Warminster, PA, USA). Jasco FT/IR 4700 Fourier Transform Infrared Spectrometer (Oklahoma City, OK, USA) was used in order to obtain the Fourier-transform infrared (FTIR) spectra.

Nafion, Chitosan, Glutaraldehyde (GA), glucose, and glucose oxidase were purchased in Merck KgaA (Darmstadt, Germany).

ZnO tetrapods (ZnO TPs) were fabricated by the environmentally friendly, catalyst-free oxidative-metal-vapor-transport method. The method includes thermal evaporation of Zn powder at 1000 °C for 1 h in the air in a ceramic crucible, followed by ZnO nucleation and ZnO TPs growth [1]. Obtained ZnO TPs powder was white, indicating the formation of ZnO from metallic Zn in presence of atmospheric O_2_.

The functionalization of ZnO TPs with Nafion was performed in accordance with our previous study [1]. In brief, the prepared ZnO TPs (3 mg) sample was mixed with Nafion (1 mL of 2% EtOH solution). Then, the droplet (8 µL) of this mixture was placed on the ITO glass (working electrode) and dried for 1 h at 65 °C. Once cooled, the electrode was cross-linked by GA (30 min, RT). After that, GOx was added (8 µL of 20 mg/mL, overnight at 4 °C) to produce a selective layer against glucose. Finally, the electrode was coated with the additional layer of Nafion polymer (5 µL of 2% Nafion in EtOH) and stored for no more than 1 day at 4 °C before use.

The chitosan-functionalized ZnO TPs electrodes were prepared in accordance with Ref. [37] (with slight modifications). Then, 8 µL of 3 mg/mL ZnO TPs in PBS solution was deposited on the ITO glass and dried for 1 h at 65 °C. The electrode then was cross-linked by GA (30 min, RT). After that, GOx was added (8 µL of 20 mg/mL, overnight at 4 °C) to produce a selective layer against glucose. Finally, the electrode was coated with the layer of chitosan biopolymer (5 µL of 0.5 wt% chitosan in 1% acetic acid) and stored for no more than 1 day at 4 °C before use.

## 3. Results and Discussion

### 3.1. Structural Properties of ZnO TPs, ZnO TPs/Chitosan, and ZnO TPs/Nafion Samples

**Figure 1:**
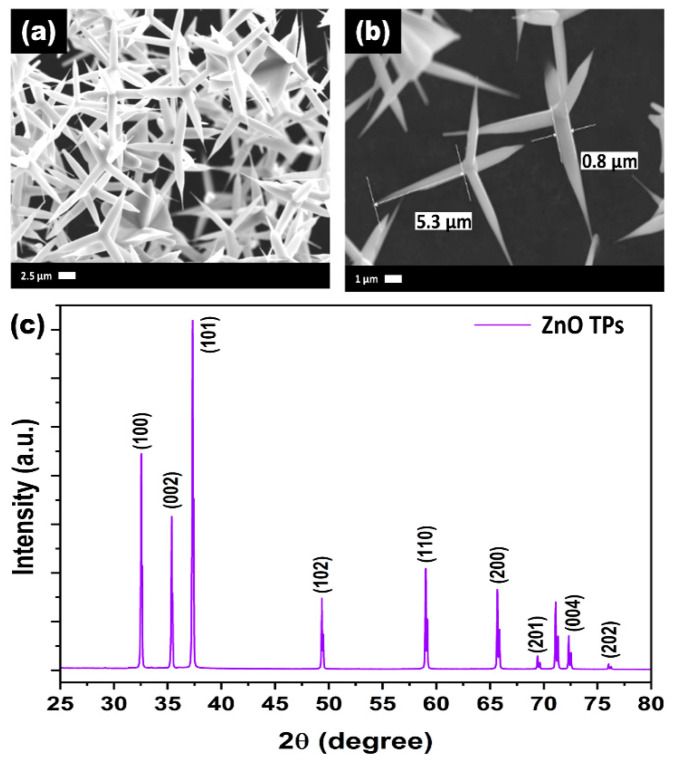
SEM images (**a**,**b**) and XRD spectra (**c**) of ZnO TPs.

**Figure 2:**
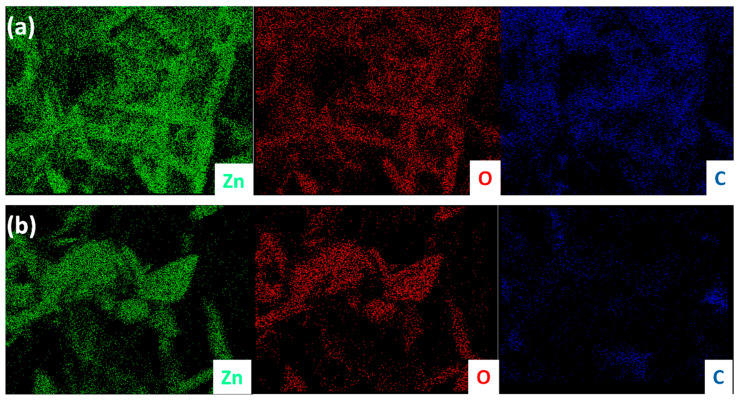
EDX mapping of ZnO TPs/chitosan (**a**) and ZnO TPs/Nafion (**b**). Green color represents zinc, red is oxygen, and dark blue is carbon.

After the successful synthesis of ZnO TPs, their morphology and structural properties was studied using SEM and XRD. Figure 1a,b represents SEM images of as-synthesized ZnO TPs consisting of 4 pods extending from one nucleus with an average pod length of about 8 ± 2 µm, and a diameter estimated at about 400 ± 150 nm. It can be seen that ZnO TPs are equally elongated into space and form massive networks with the required parameters. [38]. In order to study the phase composition of ZnO TPs, XDR measurement has been performed (Figure 1c). The peaks at 2θ = 32.57° (001), 2θ = 35.3° (002), 2θ = 37.37° (101), 2θ = 49.27° (102), 2θ = 59° (110), 2θ = 66.71° (200), 2θ = 69.45° (201), 2θ = 72.5° (004), and 2θ = 76.1° (202) confirm ZnO wurtzite structure. The lattice parameters were calculated as a = 3.25 Å, c = 5.21 Å. It should be emphasized that no additional peaks representing impurity phases were found, indicating the high purity of the prepared ZnO TPs. Furthermore, EDS mapping of the ZnO TPs/chitosan and ZnO TPs/Nafion samples (Figure 2a,b) show the content of zinc (Figure 2a,b (green maps)) and oxygen (Figure 2a,b (red maps)) in ZnO TPs, while the carbon (Figure 2a,b (dark blue maps)) content can be attributed to the chitosan and Nafion polymeric membranes. In addition, this mapping indicates homogeneous dispersion of Zn and O.

**Figure 3:**
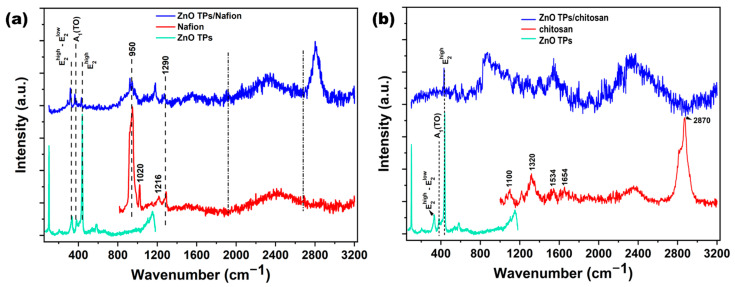
Raman spectra of ZnO TPs/Nafion (blue curve in figure (**a**)), and ZnO TPs/chitosan (blue curve in figure (**b**)) samples. Here, the light blue spectra in (**a,b**) are attributed to the ZnO TPs, the red curve in figure (**a**) is the Raman spectrum of Nafion, while the red curve in figure (**b)** is attributed to the chitosan polymer.

Raman spectroscopy is a very useful and efficient technique for determining the chemical composition of materials. In the present study, Raman spectroscopy was used to confirm ZnO TPs wurtzite structure as well as to compare spectra of ZnO TPs and ZnO TPs/chitosan, and ZnO TPs/Nafion samples. Figure 3a,b show Raman spectra of the above-mentioned samples, where the lower (light green) curve in both graphs represents the Raman spectrum on ZnO TPs with several ($E_{2}^{high}-E_{2}^{low}$, A_1_(TO), $E_{2}^{high}$) peaks that correspond to ZnO wurtzite structure [39]. The middle (red) curve in Figure 2b represents the Raman spectrum of Nafion with several vibration peaks (950, 1020, 1216 and 1290 cm^−1^) that correspond to previously proposed Nafion spectra [40]. Peaks at 950 cm^−1^ indicate the C-O-C symmetric stretching, while peaks at 1226, 1290 cm^−1^ are contributed by C-F_2_ and C-C degenerate stretches, respectively [40]. The Raman spectrum of the ZnO TPs/Nafion composite sample shows some artifacts of both materials, but with modifications, including the appearance of an intense peak at 2807 cm^−1^, which may correspond to C-H stretching.

The bands at 1654 cm^−1^ and 1591 cm^−1^ correspond to ν(C=O) and δ(NH_2_) modes which appear due to partial acetylation of NH_2_ groups in the chitosan polymer [41]. The peak at 2870 cm^−1^ is similar to the one in the Ref. [41], and may correspond to the C-H stretching [42]. In contrast to ZnO TPs/Nafion where both materials contributed, ZnO TPs/chitosan Raman spectrum does not include any artifacts of the chitosan spectrum and only includes the $E_{2}^{high}$ ZnO peak.

**Figure 4:**
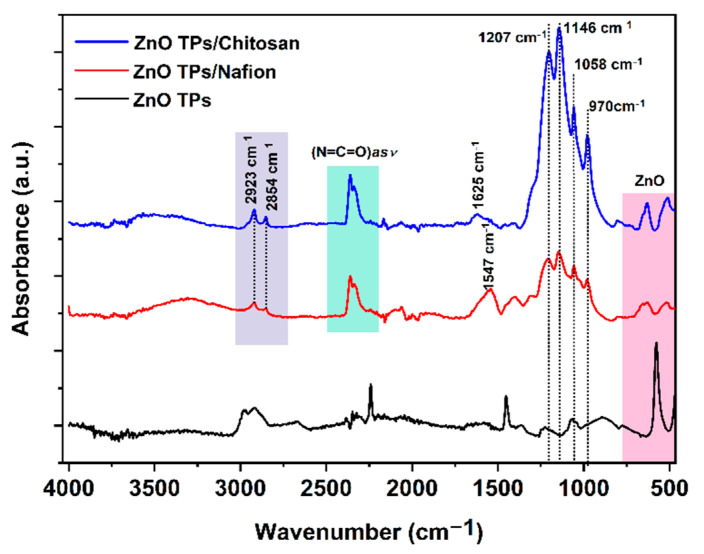
FTIR spectra of ZnO TPs, ZnO TPs/Nafion, and ZnO TPs/chitosan samples.

Fourier-transform infrared (FTIR) measurements were carried out to analyze the interactions between the ZnO TPs/chitosan and ZnO TPs/Nafion. Figure 4 represents FTIR spectra of the chitosan- and Nafion-treated ZnO TPs electrodes. The spectra of both membranes show very similar characteristic peaks all over the wavenumbers diapason including small the peak at 806 cm^−1^ (-CF_2_-CF_2_ stretch), while the peak at 970 cm^−1^ can be associated with C-O-C vibration [43]. The intense peak at 1146 cm^−1^ is contributed by the symmetric stretching of –CF_2_ groups, while peaks observed at 1058 cm^−1^ and 1207 cm^−1^ can be attributed to the symmetric and asymmetric stretching vibrations of SO^−3^ groups, respectively [43]. Peaks between 2923 and 2854 cm^−1^ can be associated with C-H symmetric and asymmetric stretching, respectively [44]. The peak at 1625 cm^−1^ indicates the presence of residual N-acetyl groups (C=O stretching of amide I) in the chitosan membrane, while the peak at 1547 cm^−1^ (ZnO TPs/Nafion curve) remained undetermined [44]. The broad peak between 3030 and 3700 cm^−1^ indicates the development of the hydrogen-bonding network [43,45].

### 3.2. Glucose Detection on ZnO TPs/Chitosan, and ZnO TPs/Nafion Samples

**Figure 5:**
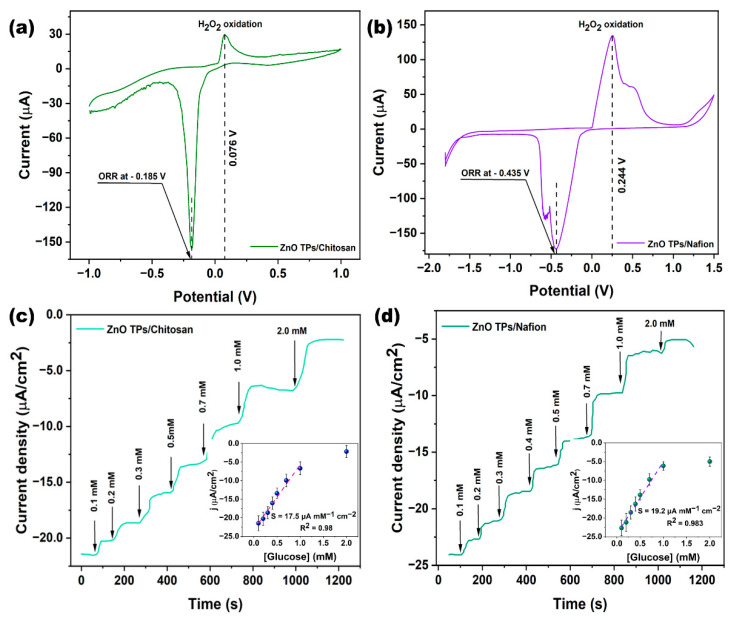
CV curves of ZnO TPs/chitosan (**a**) and ZnO TPs/Nafion (**b**) measured in PBS solution consisting of 0.3 mM of glucose. Glucose detection performances on ZnO TPs/chitosan (**c**) and ZnO TPs/Nafion electrodes (**d**). Measurements were carried out in electrochemical cell at applied potential −0.185 V for the ZnO TPs/chitosan electrode, and at −0.435 V for the ZnO TPs/Nafion electrode.

The direct electrochemistry of GOx at ZnO TPs/chitosan (Figure 5a) and ZnO TPs/Nafion (Figure 5b) has been studied by cyclic voltammetry in PBS (pH ~ 7.4) solution contained 0.3 mM of glucose. These measurements enabled to estimate the H_2_O_2_ oxidation and oxygen reduction potentials that were further applied to chronoamperometric glucose detection. One may observe that the H_2_O_2_ oxidation and oxygen reduction potentials of the ZnO TPs/chitosan electrode were 0.076 V and−0.185 V, respectively, and were significantly lower than those of the ZnO TPs/Nafion electrode (0.244 V and −0.435 V for H_2_O_2_ oxidation and oxygen reduction, respectively). The differences in redox potentials and peak currents of the ZnO TPs/chitosan and ZnO TPs/Nafion electrodes could be explained by altered mass transfers through the different micellar structures of the polymers. The detailed mechanism will be proposed in the next paragraph.

The glucose detection on ZnO TPs/chitosan and ZnO TPs/Nafion electrodes were measured by chronoamperometry measurements (Figure 5c,d). The chronoamperometry curves were taken in the cathodic mode (negative potentials), which makes it possible to eliminate interference caused by other species [1]. The stairs-like behavior of the chronoamperometry curves indicated the typical response of the GOx-treated electrodes to different concentrations of glucose that were sequentially added to the electrochemical cell. Based on these measurements, we were able to estimate important parameters regarding glucose detection on chitosan- and Nafion-treated ZnO TPs electrodes. The slopes of the plotted calibration curves (inserts Figure 5a,b) have the physical meaning of the sensitivities toward glucose, the values of the sensitivities for ZnO TPs/chitosan and ZnO TPs/Nafion electrodes (S_ZnO TPs/chitosan_=17.5 ± 1.6 µA mM^−1^ cm^−1^ and S_ZnO TPs/Nafion_ = 19.2 ± 1.3 µA mM^−1^ cm^−1^) slightly differ, indicating that the selection of the polymer (when other conditions and parameters are equal) does not play a significant role in the efficiency of glucose detection. The limits of detection (LOD) were calculated using Equation (1) [1] and were equal to 24.4 ± 2.1 µM and 22.2 ± 1.7 µM for ZnO TPs/chitosan and ZnO TPs/Nafion electrodes, respectively. The limits of quantification (LOQ) were calculated using Equation (2) and were equal to 217 ± 21 µM and 192 ± 17 µM for ZnO TPs/chitosan and ZnO TPs/Nafion electrodes, respectively.

(1) $$\mathrm{LOD}=3.3\cdot \sqrt{n}\cdot \sigma /b,$$

(2) LOQ=10σ/b,

where σ is the standard deviations of the negative control, n is the number of tests, and b is the slope of the curve from in the inset Figure 5c,d. It can be concluded that the electrodes treated with chitosan showed a lower efficiency of glucose detection since all the essential detection parameters were worse. In comparison, the LOD of the ZnO TPs/chitosan electrode was about 9% higher than that of the ZnO TPs/Nafion electrode, while the respective sensitivity was about 8% lower. The measured LOD and sensitivity of ZnO TPs electrodes modified by chitosan and Nafion point out that our electrodes are more suitable for the glucose detection in non-invasive body fluids (where glucose content is down to tens µM), while, for example, chitosan (CHI)-reduced graphene oxide (rGO)-polyaniline (PAni) electrodes reported in [46] are intended for use in blood glucose measurement, since their sensitivity lies in the mM range. Moreover, the fabrication process of chitosan (CHI)-reduced graphene oxide (rGO)-polyaniline (PAni) electrodes is much more complex than in the case of ZnO TPs/chitosan and ZnO TPs/Nafion electrodes.

### 3.3. Comparison of Chitosan and Nafion Semi-Permeable Membranes

Semipermeable membranes such as chitosan and Nafion play an important role in the production of electrodes for the electrochemical detection of analytes. Several key functions have been attributed to them, including mass transfer, enzyme trapping, and preventing their leakage [47]. It is known that hydrophilic and hydrophobic domains have naturally micellar polymer membranes which permit transport and pre-concentration of cations through the membrane structure [48]. For example, the Nafion micelle membrane is intensively used in the electrode biofunctionalization, but the Nafion-based membrane usually suffers from quite limited time stability, which results in the reduced lifetime and activity of the immobilized enzyme [49]. Moreover, Nafion usually referred to as a “forever chemical”, is neither fully destroyed in the environment nor biocompatible. On the other hand, chitosan semi-permeable membranes are made from insect shells and crustaceans, so they have excellent biocompatibility and biodegradability [50]. It is also well known for its low cost (if compared to Nafion), chemical inertness, high mechanical stability, and non-toxicity, which makes it attractive for biosensor application. Chitosan is stable and insoluble in water solutions, and similarly to the Nafion polymer, it forms micelles as the result of hydrophobic and hydrophilic segment separation [49]. In this regard, chitosan could substitute the Nafion polymer membranes in enzymatic (bio)sensors applications and other related applications.

**Figure 6:**
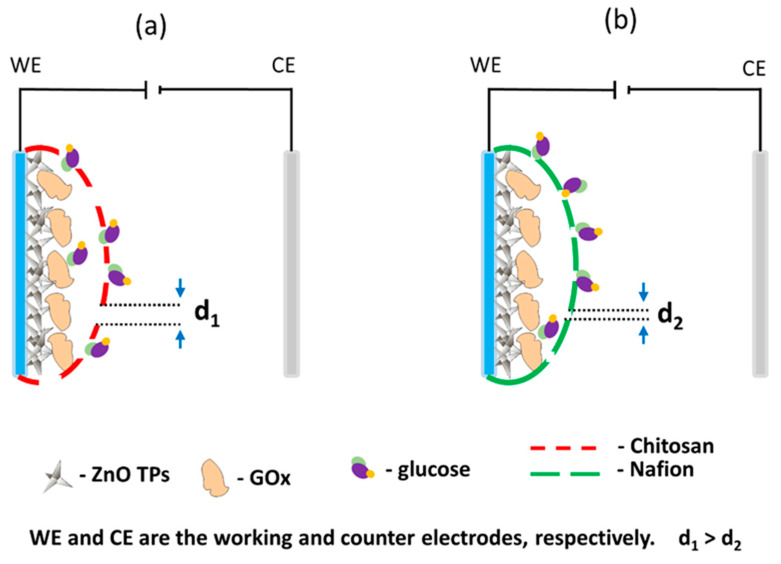
Schemes of the ZnO TPs/chitosan (**a**) and ZnO TPs/Nafion electrodes (**b**). Interpretation of the effect of micelle size on glucose transport through a semi-permeable polymer layer.

Owing to the fact that in this study, no significant improvement of (bio)sensor response was observed between ZnO TPs/chitosan and ZnO TPs/Nafion electrodes, as well as quite similar sensitivities and LODs, one may assume that the selection of the semi-permeable membrane does not play an important role in overall detection performance. Despite these similarities, there are also significant changes in CV measurements as different redox potentials. It was the oxygen reduction and H_2_O_2_ oxidation reaction on the chitosan-treated electrode that occurred at the lower potentials than for the Nafion-treated electrode, which may be explained by the different micellar structures of the semi-permeable membranes. Given the fact that electrochemical flux is highly dependent on the membrane’s micelles sizes, and the molecular weight of the analyte molecule that penetrates the membrane, it is very important to select membranes which could pass large (bio)molecules [51]. Taking into account that the glucose molecule is large enough to cause restructuring and plugging of micellar structure, that would affect glucose flux through the membrane. One may assume that the large glucose molecule would require a higher applied potential to seep through the smaller micelles, while bigger micelles would not interfere with glucose flux [49]. Thus, the lower potential of the H_2_O_2_ oxidation on the ZnO TPs/chitosan electrode can be explained by the chitosan membrane with bigger micelles than those of the ZnO TPs/Nafion membrane (d_1_ > d_2_ in Figure 6). This assumption is in good agreement with the previously reported data where the change in micelles size resulted in the changed ionic flux through the membrane [49,51].

## 4. Conclusions

In this study, ZnO TPs/chitosan and ZnO TPs/Nafion electrodes were fabricated and used for real-time glucose detection in PBS. ZnO TPs were synthesized by the environmentally friendly, catalyst-free oxidative-metal-vapor-transport method. Successful functionalization of ZnO TPs was carried out using Nafion and chitosan polymers with different micellar structures. The pods’ length of as-fabricated ZnO TPs was in the range 6–10 µm, while the average pod’s diameter was about 400 nm. The XRD measurement pointed out the high purity of the fabricated ZnO TPs powder, since no peaks that correspond to any impurities were observed. FTIR and Raman spectroscopy measurements confirm the deposition of chitosan and Nafion membranes over the ZnO TPs, which means that ZnO TPs/Nafion and ZnO TPs/chitosan samples were successfully fabricated.

The CV measurement showed that the chitosan-modified ZnO TPs electrode required the application of a lower potential for glucose oxidation (than for the ZnO TPs/Nafion electrode) due to larger micelles, which allow glucose to pass through more easily. Moreover, when other conditions and parameters are equal, the selection of polymer membrane for electrode functionalization does not play a significant role. The low LODs (24.4 µM and 22.2 µM, respectively) and LOQs (217 µM and 192 µM, respectively) of the ZnO TP/chitosan and ZnO TP/Nafion electrodes demonstrate the promise of glucose detection in non-invasive body fluids (e.g., sweat, tears).
